# Risk of dementia according to the severity of chronic periodontitis in Korea: a nationwide retrospective cohort study

**DOI:** 10.4178/epih.e2022077

**Published:** 2022-09-21

**Authors:** Seon-Rye Kim, Minkook Son, Yu-Rin Kim, Hyun-Kyung Kang

**Affiliations:** 1 Department of Healthcare Management, Youngsan University, Yangsan, Korea; 2 Department of Physiology, Dong-A University College of Medicine, Busan, Korea; 3 Department of Dental Hygiene, Silla University, Busan, Korea

**Keywords:** Dementia, Elderly, Oral health, Periodontitis

## Abstract

**OBJECTIVES:**

We investigated the risk of dementia in older adults with chronic periodontitis according to the severity of periodontitis.

**METHODS:**

Data on patients with chronic periodontitis were extracted from the National Health Insurance Service-Senior cohort database from 2002 to 2014. Among 52,728 subjects eligible for inclusion, 11,953 subjects had newly diagnosed mild chronic periodontitis (MCP), and 40,775 subjects had newly diagnosed severe chronic periodontitis (SCP). Two 1:1 propensity score matched cohorts were created with 8,624 patients each in the MCP and SCP groups. To analyze the risk of dementia, a Cox proportional-hazard model was used to calculate hazard ratios with 95% confidence intervals (CIs).

**RESULTS:**

In the Kaplan-Meier curve, the disease-free probability was significantly lower in the SCP group than in the MCP group (p for log-rank=0.001). In the multivariable-adjusted model, the HR for the occurrence of dementia in the SCP group compared to the MCP group was 1.15 (95% CI, 1.04 to 1.27; p=0.009). A subgroup analysis revealed a significant association between dementia and the severity of periodontitis, especially in subjects who were male, aged ≥70 years, and had comorbidities.

**CONCLUSIONS:**

Reducing the severity of chronic periodontitis can help to reduce the risk of dementia. Therefore, it is necessary to aggressively conduct early dementia-prevention programs for males under the age of 70 that include dental health to prevent the progression of periodontitis from mild to severe.

## INTRODUCTION

Scholars worldwide have been discussing issues associated with the increase in human longevity [1]. As people age, their chances of developing dementia increase. It has also been estimated that the population with dementia will double by 2030 and triple by 2050 [2]. Patients with dementia experience memory loss, language difficulties, impaired problem solving and other cognitive disabilities, and progressively lose their ability to maintain daily activities, increasing the societal burden [3,4]. The early stages of dementia are difficult to detect and prevent. Several studies have focused on the risk factors of dementia to address the need for early diagnosis, treatment, and prevention [5-8]. One retrospective cohort study with a 10-year follow-up period suggested that older age, female sex, diet, alcohol consumption, smoking status, obesity, diabetes mellitus, hypertension, heart disease, stroke, depression, intracranial injury, and mild cognitive impairment are risk factors for dementia [6]. However, there are still no definitive intervention strategies to effectively reduce the prevalence of dementia.

The present study aims to contribute to the goal of identifying and managing the risk factors associated with dementia. One such risk factor is chronic periodontitis [8]. While chronic periodontitis is an important health issue for the general population, it is highly prevalent among older adults [9]. The 2016 to 2018 Korea National Health and Nutrition Examination Survey results showed that 47.7% of adults ≥ 60 years old had either moderate or severe periodontitis [10]. Other studies showed that chronic periodontitis was strongly associated with dementia [11-14]. In addition, a Korean study found that people with natural dentition had better cognitive ability than those with dentures [14]. Among other environmental risk factors, it is reasonable to consider chronic periodontitis a link to dementia because of the relationship between oral health conditions and the host’s immune system. Several systemic diseases have been associated with periodontitis, including cardiovascular disease, diabetes, and cancer [15].

There have been numerous studies of periodontitis in older adults. Previous research compared elderly patients with periodontitis to those without periodontitis to determine whether periodontitis was a risk factor for the development of dementia. Most researchers view periodontitis as a risk factor for the development of dementia, yet debate continues internally over the correlation between the 2 conditions. Therefore, our research was designed to analyze the severity of periodontitis, not simply to identify whether a patient has or does not have the condition. Subsequently, the data collected on patients with periodontitis were divided into groups: mild and severe disease. The propensity score matching (PSM) method was used to compare patient data from the mild and severe groups. Attempts were made to align data as closely as possible to create similar matches, with the exception of the severity of periodontitis.

The aims of this study were: (1) to determine the prevalence of dementia according to the severity of existing chronic periodontitis and (2) to investigate the relationship between dementia and chronic periodontitis in Korea’s older adult population using the Korean National Health Insurance Service (NHIS) database, a representative source of health data on all Korean citizens.

## MATERIALS AND METHODS

### Subject selection

**Figure 1.**
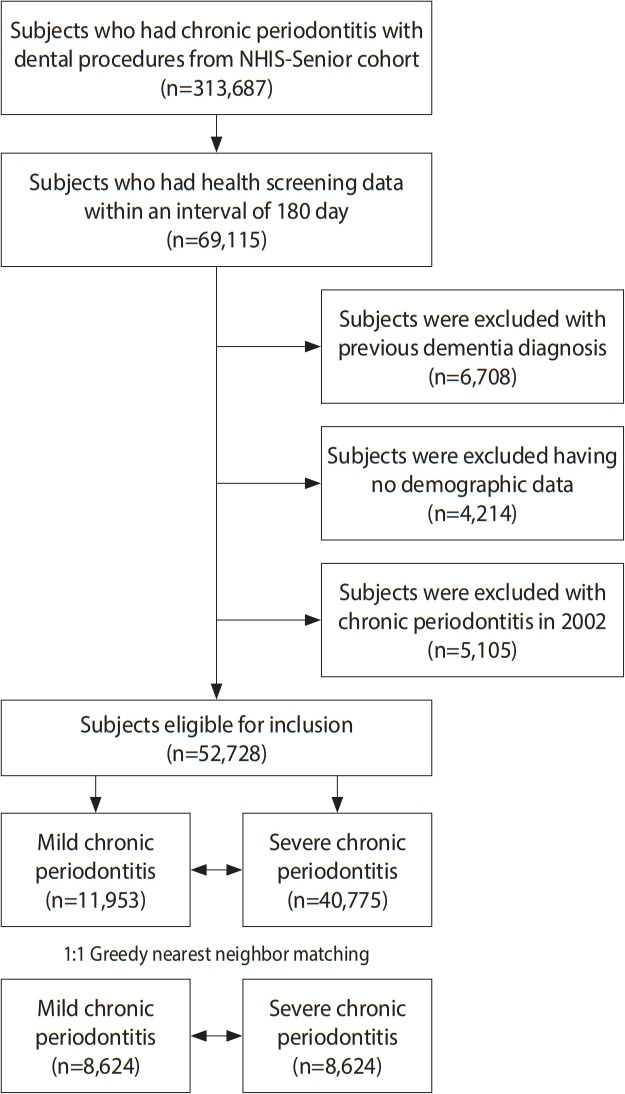
Flow of the study population. NHIS, National Health Insurance Service.

The study population was derived from the NHIS-Senior cohort. In Korea, the NHIS covers nearly all forms of healthcare for all Koreans [16]. From its claims database, the NHIS provides data for research purposes that includes information on inpatient and outpatient hospital use, drug prescriptions, death dates, and results from health screening examinations. The NHIS-Senior cohort databases include diagnoses, drug prescriptions, treatments, and mandatory health screening examination results for those aged ≥ 60 years [17]. Among 313,687 subjects from the NHIS-Senior cohort with chronic periodontitis who underwent dental procedures, there were 69,115 subjects who had health screening data within an interval of 180 days. We excluded 6,708 subjects who were previously diagnosed with dementia, and 5,105 subjects who were diagnosed with chronic periodontitis in 2002. We also excluded 4,214 subjects with incomplete demographic data. Among the 52,728 subjects eligible for inclusion, 11,953 subjects had newly diagnosed mild chronic periodontitis (MCP) and 40,775 subjects had newly diagnosed severe chronic periodontitis (SCP). After 1:1 greedy nearest-neighbor matching, the final study population consisted of 8,624 subjects in the mild group and 8,624 subjects in the severe group. Starting from the index date, the subjects were followed up until the date of dementia diagnosis, date of death, or December 31, 2015, whichever came first (Figure 1).

### Key variables

The NHIS defined chronic periodontitis using the 10th revision of International Classification of Diseases (ICD-10) codes by the World Health Organization (K051 and K053). Subjects who received dental procedures such as scaling, root planing, and subgingival curettage were classified in the MCP group. Subjects who received dental procedures such as tooth extraction, periodontal flap surgery, bone graft for alveolar bone defects, or guided tissue regeneration were classified in the SCP group [18]. Finally, the severity of periodontitis was classified as MCP or SCP.

The covariates included matching variables such as age, sex, income level (quartile groups), smoking status, alcohol consumption, regular exercise, hypertension (I10-I15), diabetes (E10-E14), dyslipidemia (E78), heart disease (I20-I25), cerebrovascular disease (I60-I69), depression (F32-F33), and Charlson comorbidity index (CCI) scores. The covariates also included adjusted variables such as body mass index (BMI), systolic blood pressure, diastolic blood pressure, fasting blood glucose levels, and total cholesterol levels (Supplementary Matreial 1).

The CCI was initially developed as a scoring system for medical inpatients and was designed to rank comorbidities into specific risk groupings by assigning type and severity scores to a range of specific illnesses [19]. At the start of the follow-up period, a baseline CCI score was calculated based on preexisting diseases including myocardial infarction, congestive heart failure, peripheral vascular disease, cerebrovascular disease, dementia, chronic pulmonary disease, connective tissue disease, peptic ulcer, mild liver disease, diabetes with and without complications, paraplegia or hemiplegia, renal disease, any primary or metastatic cancer, moderate or severe liver disease, and AIDS [20].

### Outcome

The NHIS defined dementia using the ICD-10 codes F00, F01, and F03. In addition, dementia patients were classified as those with ≥ 1 admission or ≥ 2 outpatient visits with ≥ 1 prescription of dementia drugs (Supplementary Material 1).

### 1:1 Propensity score matching

To ensure the homogeneity of the 2 groups, age, sex, income level, smoking status, alcohol consumption, regular exercise, hypertension, diabetes, dyslipidemia, heart disease, cerebrovascular disease, depression, and CCI were applied using the 1:1 PSM method. PSM analysis was used on the sampled cohorts to determine selection bias and the presence of potential confounding variables. In addition, the standardized difference in the PSM analysis was matched to less than 0.025, ensuring the homogeneity of the 2 groups.

### Statistical analysis

The NHIS-Senior cohort database was analyzed using R 3.6.0 (https://www.r-project.org/) and SAS version 9.4 (SAS Institute Inc., Cary, NC, USA). The adjusted variables of the baseline characteristics, before and after PSM, were compared using the Student t-test and the chi-square test. A Kaplan-Meier curve was generated for dementia risk analysis and a log-rank test was performed. The incidence rate of dementia was presented in units of 1,000 person-years for the total follow-up period of the MCP and SCP groups. To analyze the risk of dementia, the Cox proportional-hazards model was used to calculate the hazard ratio (HR) and 95% confidence intervals (CIs). In model 1, age, sex, income level, hypertension, diabetes, dyslipidemia, heart disease, cerebrovascular disease, depression, smoking, alcohol consumption, regular exercise, and CCI were adjusted. In model 2, BMI, blood pressure, fasting blood glucose level, and total cholesterol level were adjusted in addition to the characteristics in model 1. The p-values < 0.05 were considered to indicate statistical significance.

### Ethics statement

This study was approved by the Institutional Review Board of Youngsan University (YSUIRB-202103-HR-085-02).

## RESULTS

### Demographic characteristics according to the severity of chronic periodontitis

A total of 17,248 study subjects were followed for a median period of 6.6 years, during which 1,514 cases of dementia occurred (8.7%). After collecting demographic data according to the presence of MCP or SCP, but before conducting PSM, there were 11,953 subjects with MCP and 40,775 subjects with SCP. After PSM, there were 8,624 subjects in both groups. The standardized mean difference for age was < 0.1 after PSM. The proportion of males to females was higher in the SCP group before PSM, but was equal after PSM. After PSM, smoking and alcohol consumption, regular exercise, hypertension, diabetes, dyslipidemia, heart disease, cerebrovascular disease, depression, and CCI results were all confirmed equally with a decreased standardized mean difference. Data from health screenings were used as adjusted variables, showing significant differences in systolic blood pressure, diastolic blood pressure, and fasting blood glucose levels after PSM. The incidence of dementia was significantly higher in the SCP group both before and after PSM (Table 1).

### Comparison of dementia risk according to the severity of chronic periodontitis

**Figure 2.**
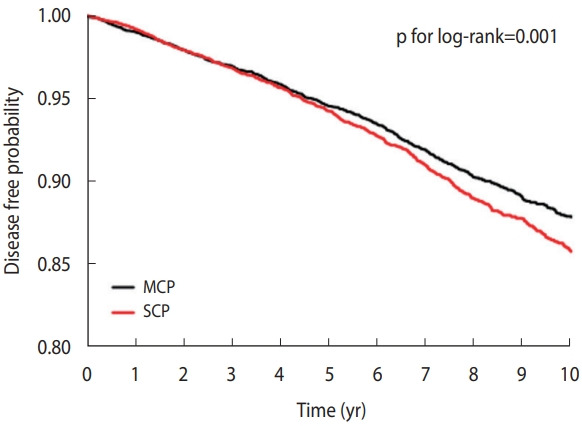
Kaplan-Meier curve for dementia associated with chronic periodontitis. MCP, mild chronic periodontitis; SCP, severe chronic periodontitis.

The disease-free probability decreased significantly in the SCP group compared to the MCP group in the Kaplan-Meier curve. In the log-rank test, the p-value was statistically significant at 0.001 (Figure 2). Of the 17,248 study subjects, 804 cases of dementia occurred in the SCP group and 710 cases in the MCP group. The incidence rate (per 1,000 person-years) was higher in the SCP group (14.10) than in the MCP group (12.46).

Dementia was significantly associated with the severity of periodontitis in the crude and multivariable-adjusted Cox proportional hazards model. The crude HR for the occurrence of dementia in the SCP group compared to the MCP group was 1.13 (95% CI, 1.02 to 1.25). The adjusted HR in model 1 was 1.14 (95% CI, 1.03 to 1.26). The adjusted HR in model 2 was 1.15 (95% CI, 1.04 to 1.27) (Table 2).

### Subgroup analysis according to sex and age

In the subgroup analysis by sex, the incidence rate of dementia in the SCP group was higher than in the MCP group, regardless of sex. However, the adjusted HR for dementia in the female group was not significant. The adjusted HR for dementia in males in the SCP group compared to the MCP group was 1.27 (95% CI, 1.04 to 1.54) in model 1 and 1.26 (95% CI, 1.04 to 1.53) in model 2. The subgroup analysis by age divided subjects into groups > 70 years and < 70 years. The incidence rate of dementia in the SCP group was higher than that of the MCP group, regardless of age. The adjusted HR for dementia in the SCP group compared to the MCP group was 1.13 (95% CI, 1.00 to 1.27) in model 1 and 1.13 (95% CI, 1.00 to 1.28) in model 2 in the subjects who were > 70 years of age (Table 2).

## DISCUSSION

The purpose of this study was to identify the prevalence of dementia in older Korean adults according to the severity of chronic periodontitis and to investigate the relationship between the 2 medical conditions. Dementia is a multi-pathogenic syndrome characterized by accumulated cognitive impairments from multiple cognitive and behavioral domains that impair the functional abilities of a patient. Although it can begin at any age, it mainly affects the elderly [1]. The exact causes of and treatments for dementia have not yet been identified [21]. Furthermore, numerous studies on diseases such as dementia and Alzheimer’s disease are underway as global populations age [5,8,12,13,15]. Despite extant research, there are insufficient findings available to elucidate the causal relationship. Unfortunately, no cure for dementia currently exists, so many researchers are focusing their work on the risk factors for dementia [22], including chronic periodontitis [23-25]. Most researchers view periodontitis as a risk factor for dementia, and several studies have examined whether the presence or absence of chronic periodontitis is related to dementia or the risk of developing dementia [26] including Alzheimer’s disease [27]. While one study found that periodontal status deteriorated with the progress of Alzheimer’s disease [13], several studies also showed that patients with periodontitis had an increased risk of dementia compared to a control group [24-27]. Despite these findings, the association between chronic periodontitis and dementia is still not certain. This uncertainty is due to limitations in previous studies, such as small sample sizes [28,29], a focus on a specific population [24,28,29], inadequate adjustment for confounding factors [28], lack of sensitivity analysis [24-27], and unclear cases of periodontitis [26,27,30].

To properly investigate the effects of chronic periodontitis on dementia, it is necessary not only to check the presence or absence of chronic periodontitis but also to check the data of patients with chronic periodontitis to determine and classify its severity. In this study, the data of patients with periodontitis were divided into groups with MCP or SCP. To reduce bias, data were first sorted by PSM, and then analyzed. Additionally, the data were suitable for comparing and analyzing both groups. As shown in a KaplanMeier curve, the disease-free probability in the MCP group was higher as time passed; meanwhile, as the intensity of the SCP group progressed, the correlation with dementia also increased. Since it has been reported that the 2 most prominent risk factors for dementia are advanced age and sex [31], this study also analyzed its results according to sex and age. In this study, the incidence of dementia was significantly higher in the SCP group than in the MCP group for all ages (p< 0.01), and there was a significant difference in the incidence of dementia according to the severity of periodontal disease among people over age 70. As for sex, there was a significant difference in the incidence of dementia between males and females. One previous study also reported that oral hygiene status was related to cognitive function in the elderly [11]. In a study to predict cognitive decline in the elderly, the incidence of tooth loss, periodontal disease, and dental caries increased with age [8]. A 4-year prospective study also reported a significant association between increasing age and cognitive impairment in elderly males, as well as observing that tooth loss could be a predictor or risk factor for dementia [12]. The present study has similarities with other research findings, including a meaningful difference in the incidence of dementia in individuals aged ≥ 70 years, highlighting the need for policies that support early treatment of periodontitis, especially in adult males under the age of 70. Since this research concerns dementia in relation to the severity of periodontal disease, it is possible to prevent or manage the risk of dementia in patients while also preventing further deterioration of periodontitis. Alzheimer’s disease accounts for 60-80% of all cases of dementia [32]. Alzheimer’s disease is more common in females than in males, in those with a lower level of education, in those who have immediate family members with dementia, and with increasing age [21]. Females reportedly have a higher risk of developing dementia from Alzheimer’s disease, while males have a higher risk of vascular dementia [31]. In this study, dementia was found to increase in elderly males more than in females.

It has recently been found that inflammation plays an important role in the development and progression of Alzheimer’s disease [30], and efforts have been made to control and prevent the risk factors for Alzheimer’s related to systemic inflammation [5]. Periodontitis is a highly prevalent chronic inflammatory disease, and periodontal pathogens induce an immune-inflammatory response in the host that destroys periodontal tissue. This tissue destruction eventually causes tooth loss. The immune-inflammatory response generated by periodontitis also increases the host’s sensitivity to various diseases. The local inflammation of periodontitis induces a systemic inflammatory state through the transmission of bacteria or inflammatory cytokines [33]. In the elderly, periodontitis can induce an inflammatory state and accelerate neurodegenerative diseases [34]. Notably, other studies have reported that the systemic inflammatory response induced by periodontitis increases the risk of Alzheimer’s disease [35,36]. Considering the evidence so far, the relationship between periodontitis and dementia is very strong [11,12,37]. Periodontitis, a representative oral chronic inflammatory disease that ultimately causes tooth loss, can accelerate the progression of neurodegenerative diseases by exacerbating the inflammatory state [9]. In particular, one study reported that worsening neuroinflammatory responses in elderly animals induced depressive-like behaviors and cognitive deficits like those found in Alzheimer’s disease [38]. *Porphyromonas gingivalis* lipopolysaccharide, found in the brains of mouse models with Alzheimer’s disease, has been shown to accelerate neuroinflammation, making it highly susceptible to terminal inflammation [39]. In addition, when the serum markers of *P. gingivalis*, a periodontal pathogen, increased in patients ≥ 60 years, cognitive abilities such as word memory were found to be low [25]. Based on these observations, we suggest that inflammation plays an important role in the development and progression of dementia and that it is necessary to prevent dementia by controlling the various risk factors related to inflammatory reactions.

The American Academy of Periodontology (AAP) classifies periodontitis as slight, moderate, or severe based on clinical attachment loss [40]. A limitation of this study was that the NHIS data did not include details of oral examinations, so our classifications of periodontal disease were based on the treatment plans and may differ from the AAP classification system. However, as there has been no analysis of dementia using a national database and based on the severity of periodontitis, the data from our research should be helpful to others who study dementia. Another limitation is that causality cannot be guaranteed due to the retrospective cohort study design. Nevertheless, we tried to increase the reliability of the results by setting the washout period at 1 year when defining new patients with chronic periodontitis, and excluding those diagnosed with dementia before being diagnosed with chronic periodontitis. Furthermore, we conducted a mediation analysis to check the mediating effect of chronic periodontitis on dementia and confirmed that chronic periodontitis is associated with dementia as a direct effect without mediating effect (Supplementary Materials 2 and 3).

Despite advances in early diagnosis and easing the pace of disease progression, there is still a great deal of interest in research to prevent dementia because no definitive treatment has been found. Since the number of patients with dementia increases with age, the topic gains more attention as societies age. Recognizing that the inflammatory response of periodontitis is highly correlated with dementia, the management of dental plaque (the local cause of periodontitis) should be prioritized. Control of dental plaque bacteria can prevent periodontitis and help maintain and improve oral health by inhibiting the production of inflammatory cytokines.

In conclusion, the incidence of dementia was significantly higher in the SCP group than in the MCP group for all ages, in males than in females, and in those over 70 years of age than in younger subjects. Oral care for the elderly is generally important, but to prevent severe periodontitis that can lead to dementia, it is necessary to focus early dementia prevention on the oral care of males under the age of 70 to prevent mild periodontitis from progressing to severe periodontitis. In addition, future investigations according to the type of dementia (i.e., Alzheimer’s dementia vs. vascular dementia) are recommended as well as studies of the relationship between the masticatory function and oral health (e.g., dental caries) of the elderly and dementia.

## Figures and Tables

**Table 1.** Baseline characteristics of the study population

Characteristics		Before PSM (n=52,728)				After PSM (n=17,248)			
		MCP (n=11,953)	SCP (n=40,775)	Standardized mean difference	p-value	MCP (n=8,624)	SCP (n=8,624)	Standardized mean difference	p-value
Demographics (matching variables)									
	Age (yr)	71.6±5.0	70.5±5.4	0.20	-	70.9±4.8	71.0±4.9	-0.02	-
	Sex, male	4,897 (41.0)	20,013 (49.1)	-0.16	-	2,942 (34.1)	2,942 (34.1)	0.00	-
	Income level (quartile)			0.15	-			0.02	-
	1st	1,918 (16.0)	7,704 (18.9)	-	-	1,316 (15.3)	1,268 (14.7)	-	-
	2nd	1,911 (16.0)	8,050 (19.7)	-	-	1,418 (16.4)	1,534 (17.8)	-	-
	3rd	3,115 (26.1)	10,955 (26.9)	-	-	2,332 (27.0)	2,414 (28.0)	-	-
	4th	5,009 (41.9)	14,066 (34.5)	-	-	3,558 (41.3)	3,408 (39.5)	-	-
	Smoking	750 (6.3)	5,733 (14.1)	-0.21	-	396 (4.6)	396 (4.6)	0.00	-
	Drinking alcohol	2,397 (20.1)	10,368 (25.4)	0.12	-	1,325 (15.4)	1,325 (15.4)	0.00	-
	Regular exercise	1,184 (9.9)	4,633 (11.4)	0.05	-	537 (6.2)	537 (6.2)	0.00	-
	Hypertension	9,021 (75.5)	28,659 (70.3)	-0.12	-	6,655 (77.2)	6,655 (77.2)	0.00	-
	Diabetes	6,040 (50.5)	17,379 (42.6)	-0.16	-	4,236 (49.1)	4,236 (49.1)	0.00	-
	Dyslipidemia	7,452 (62.3)	19,162 (47.0)	-0.31	-	5,225 (60.6)	5,225 (60.6)	0.00	-
	Heart disease	3,946 (33.0)	9,621 (23.6)	-0.21	-	2,474 (28.7)	2,474 (28.7)	0.00	-
	Cerebrovascular disease	2,906 (24.3)	7,044 (17.3)	-0.17	-	1,676 (19.4)	1,676 (19.4)	0.00	-
	Depression	2,607 (21.8)	5,983 (14.7)	-0.19	-	1,473 (17.1)	1,473 (17.1)	0.00	-
	Charlson Comorbidity Index	3.7±2.9	2.7±2.6	0.38	-	4.3±3.0	4.3±2.8	0.01	-
Health screening data (adjusting variables)									
	BMI	24.1±3.1	23.9±3.1	-	<0.001	24.2±3.1	24.2±3.2	-	0.640
	Systolic blood pressure	130.9±16.6	132.0±17.6	-	<0.001	131.7±16.8	132.2±17.2	-	0.030
	Diastolic blood pressure	78.4±10.3	79.4±10.8	-	<0.001	78.9±10.4	79.3±10.6	-	0.002
	Fasting blood glucose	102.1±26.9	103.2±32.0	-	<0.001	101.8±26.0	103.0±28.5	-	0.002
	Total cholesterol	196.7±38.8	197.5±38.7	-	0.060	198.7±38.9	197.9±39.6	-	0.210
Outcome									
	Dementia	966 (8.1)	4,594 (11.3)	-	<0.001	710 (8.2)	804 (9.3)	-	0.010

Values are presented as mean±standard deviation or number (%). PSM, propensity score matching; MCP, mild chronic periodontitis; SCP, severe chronic periodontitis; BMI, body mass index.

**Table 2.** Association between chronic periodontitis and dementia

Variables	Dementia events (n)	Follow-up duration (person-yr)	Incidence rate (per 1,000 person-yr)	Crude	p-value	Model 1^1^	p-value	Model 2^2^	p-value
All (n=17,248)									
MCP	710	57,005	12.46	1.00 (reference)		1.00 (reference)		1.00 (reference)	
SCP	804	57,009	14.10	1.13 (1.02, 1.25)	0.01	1.14 (1.03, 1.26)	0.01	1.15 (1.04, 1.27)	0.01
Male (n=5,884)									
MCP	183	21,524	8.50	1.00 (reference)		1.00 (reference)		1.00 (reference)	
SCP	230	21,464	10.72	1.26 (1.04, 1.53)	0.02	1.27 (1.04, 1.54)	0.02	1.26 (1.04, 1.53)	0.02
Female (n=11,364)									
MCP	527	35,481	14.85	1.00 (reference)		1.00 (reference)		1.00 (reference)	
SCP	574	35,545	17.11	1.09 (0.97, 1.22)	0.17	1.09 (0.97, 1.23)	0.14	1.10 (0.98, 1.24)	0.11
Age <70 yr (n=6,583)									
MCP	225	30,569	7.36	1.00 (reference)		1.00 (reference)		1.00 (reference)	
SCP	264	30,755	8.58	1.16 (0.97, 1.39)	0.09	1.18 (0.99, 1.42)	0.06	1.18 (0.99, 1.41)	0.06
Age ≥70 yr (n=10,665)									
MCP	485	26,436	18.35	1.00 (reference)		1.00 (reference)		1.00 (reference)	
SCP	540	26,254	20.57	1.13 (1.00, 1.28)	0.05	1.13 (1.00, 1.27)	0.05	1.13 (1.00, 1.28)	0.05

Values are presented as hazard ratio (95% confidence interval). MCP, mild chronic periodontitis; SCP, severe chronic periodontitis.

1 Aadjusted for age, sex, income level, hypertension, diabetes, dyslipidemia, heart disease, cerebrovascular disease, depression, smoking, alcohol consumption, regular exercise, and Charlson comorbidity index.

2 Adjusted for age, sex, income level, hypertension, diabetes, dyslipidemia, heart disease, cerebrovascular disease, depression, smoking, alcohol consumption, regular exercise, Charlson comorbidity index, body mass index, blood pressure, fasting blood glucose, and total cholesterol levels.

## SUPPLEMENTARY MATERIALS

Supplementary Material 1. Definitions of diseases by disease classification codes and insurance claim codes

Supplementary Material 2 Schematic causal diagram for mediation analysis

Supplementary Material 3 Results of mediation analysis between chronic periodontitis and the clinical variables for dementia
