# Supplementary material for: Risk of dementia according to the severity of chronic periodontitis in Korea: a nationwide retrospective cohort study
Source: Epidemiol Health. 2022 Sep 21;44:e2022077. doi: 10.4178/epih.e2022077 (PMC9849849; doi:10.4178/epih.e2022077)
Supplement: Supplementary Material 1. — Definitions of diseases by disease classification codes and insurance claim codes [file epih-44-e2022077-suppl1.docx]

**Supplementary Material 1. Definitions of diseases by disease classification codes and insurance claim codes**

| **Disease** | **ICD-10 codes** | **Claim codes** |
| --- | --- | --- |
| Mild chronic periodontitis | K051, K053 | Dental procedures including U2232, U2233, U2240, U1010, |
| Severe chronic periodontitis | K051, K053 | Dental procedures including U4412, U4413, U1051, U1052, U1071, U1072, U1081, U1082, U1083, UY101 |
| Hypertension | I10 – I15 | Outpatient ≥ 1 |
| Diabetes | E10 – E14 | Outpatient ≥ 1 |
| Dyslipidemia | E78 | Outpatient ≥ 1 |
| Heart disease | I20 – I25 | Outpatient ≥ 1 |
| Cerebrovascular disease | I60 – I69 | Outpatient ≥ 1 |
| Depression | F32 – F33 | Outpatient ≥ 1 |
| Dementia | F00, F01, F03 | Admission ≥1 or outpatient ≥2 with prescription for dementia drugs ≥1 |

ICD-10, Tenth Revision of International Classification of Diseases
