# Supplementary figures and images for: Risk of dementia according to the severity of chronic periodontitis in Korea: a nationwide retrospective cohort study
Source: Epidemiol Health. 2022 Sep 21;44:e2022077. doi: 10.4178/epih.e2022077 (PMC9849849; doi:10.4178/epih.e2022077)

**Supplementary Material 2. Schematic causal diagram for mediation analysis**


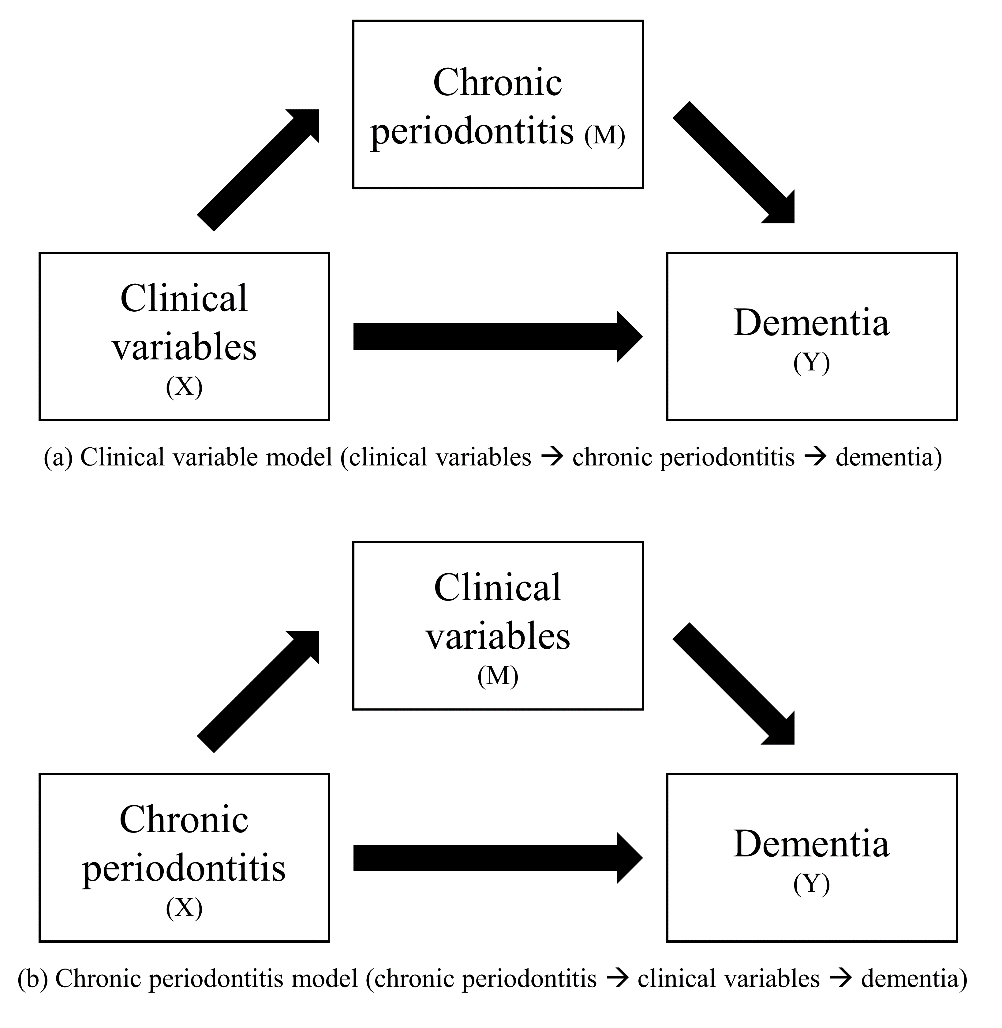

Supplement: Supplementary Material 2 — Schematic causal diagram for mediation analysis [file epih-44-e2022077-suppl2.docx]
