# Supplementary material for: Risk of dementia according to the severity of chronic periodontitis in Korea: a nationwide retrospective cohort study
Source: Epidemiol Health. 2022 Sep 21;44:e2022077. doi: 10.4178/epih.e2022077 (PMC9849849; doi:10.4178/epih.e2022077)
Supplement: Supplementary Material 3 — Results of mediation analysis between chronic periodontitis and the clinical variables for dementia [file epih-44-e2022077-suppl3.docx]

**Supplementary Material 3. Results of mediation analysis between chronic periodontitis and the clinical variables for dementia**

| **Clinical variables** | ***P*-value for interaction** | ***P*-value for ACME in clinical variable model (a)*** | ***P*-value for ACME in chronic periodontitis model (b)*** |
| --- | --- | --- | --- |
| Sex | 0.16 | 0.67 | 0.98 |
| Age | 0.99 | 0.74 | 0.98 |
| Hypertension | 0.71 | 0.27 | 0.99 |
| Diabetes | 0.27 | 0.76 | 0.97 |
| Dyslipidemia | 0.63 | 0.33 | 0.99 |
| Heart disease | 0.49 | 0.80 | 0.98 |
| Cerebrovascular disease | 0.50 | 0.86 | 0.93 |
| Depression | 0.34 | 0.85 | 0.95 |

ACME: average causal mediation effect

*These mediation models were adjusted for age, sex, income level, hypertension, diabetes, dyslipidemia, heart disease, cerebrovascular disease, depression, smoking, alcohol consumption, regular exercise, Charlson Comorbidity Index, body mass index, blood pressure, fasting blood glucose and total cholesterol levels.
